# Pharmacological Treatments in Gambling Disorder: A Qualitative Review

**DOI:** 10.1155/2014/537306

**Published:** 2014-05-18

**Authors:** Matteo Lupi, Giovanni Martinotti, Tiziano Acciavatti, Mauro Pettorruso, Marcella Brunetti, Rita Santacroce, Eduardo Cinosi, Giuseppe Di Iorio, Marco Di Nicola, Massimo Di Giannantonio

**Affiliations:** ^1^Department of Neuroscience and Imaging, University “G. d'Annunzio”, Chieti, Italy; ^2^Department of Psychiatry, Drug Addiction Unit, Catholic University Medical School, Rome, Italy

## Abstract

Gambling disorder (GD) is a psychiatric condition associated with both social and family costs; DSM-5 currently includes GD among addictive disorders. Despite the high burden of this condition, to date there are no treatment guidelines approved by Food and Drug Administration (FDA). Purpose of this paper is to offer a qualitative overview about the different pharmacologic agents used for the treatment of GD. Our analysis, conducted on a final selection of 75 scientific papers, demonstrates that a variety of pharmaceutical classes have been utilised, with different results. Published data, although limited by brief duration of the studies and small number of enrolled subjects, shows mixed evidence for serotonergic antidepressants, opioid antagonists, and mood stabilizers. Other compounds, such as glutamatergic agents and psychostimulants, deserve further studies.

## 1. Introduction

Gambling disorder (GD) or pathological gambling is a psychiatric condition characterized by persistent and recurrent maladaptive gambling behaviour. Gambling disorder affects 0.2–5.3% of adults worldwide; the devastating consequences of this behavioural disturbance often entail severe damage to the lives of patients and their families. Previously considered among impulse control disorders, the new DSM-5 considers GD as a behavioural addiction, sharing neurobiological and clinical similarities with substance use disorders [1].

GD is classified under the “Addictive Disorders” section, reflecting the common substrate of addiction and highlighting recent finding on its pathophysiology and treatment. In addition, like substance-related disorders, GD presents phenomena of tolerance, withdrawal, and craving. Its onset is usually in early adolescence in men and between the ages of 20 and 40 years in women [2, 3].

Pathological gamblers show specific temperamental and character dimensions as novelty seeking (NS) and self-transcendence (ST), with a growing dependence on gambling, an increase in the frequency and time spent playing, a rise in the amount of money spent attempting to recover from financial losses (e.g., investing more than the budget allows by borrowing money), and a neglect of the commitments that life requires. GD patients suffer a significant impairment in social and professional functioning [4, 5].

The majority of pathological gamblers do not seek treatment: most commonly, in fact, there are family members pressuring the affected relative in order to start a therapy.

Although GD is a frequent disorder that can significantly compromise patients' quality of life, nowadays there are no treatment guidelines approved by the Food and Drug Administration (FDA). Therefore, pharmacologic therapies should be focused on clinical dimensions (i.e., impulsivity, compulsivity, and anhedonia) or on the contingent comorbid psychiatric disorders and individualised in relation to the specific characteristics of the patient [6, 7].

In recent years, several controlled clinical trials have been conducted on a variety of pharmaceutical classes, establishing an evidence-based background for the disease [8].

The aim of this paper is to review the role of different pharmacologic agents used for the treatment of GD, in order to help guide clinical decisions according to latest data.

## 2. Methods

We searched PubMed (http://www.ncbi.nlm.nih.gov/pubmed) to identify published meta-analysis, reviews, open-label trials, randomized double-blind trials, placebo-controlled trials, and case reports written in English, focusing on the pharmacotherapy of pathological gambling. The following keywords were used: gambling disorder, pathological gambling, pharmacotherapy, and treatment.

The search was conducted on October 16th, 2013, and yielded a total of 398 results. By reading titles and abstracts we excluded 323 articles from total records, in order to consider available abstracts and clinical and pharmacological trials. The full texts of the remaining 75 papers have been analysed to perform a qualitative synthesis, reported in this overview. In addition, we have searched Scopus, Google Scholar, and PsychInfo to identify any other study missed by the previous analysis. No further study has been evidenced using the same keywords.

## 3. The Different Pharmacological Approaches

The different pharmacological approaches currently considered for GD derive from the main psychopathological and phenomenological perspectives of the disorder itself. In fact, GD may be considered as belonging to the obsessive-compulsive disorder spectrum, as a behavioural addiction or as the result of an emotional dysregulation closely related to mood disorders [9, 10].

In the first case, pharmacological approach is based on antiobsessive or antidepressant drugs, in order to improve serotoninergic transmission. Drug dose is usually medium-high and the treatment lasts longer than in depression. Controlled trials have shown positive results, in particular for fluvoxamine, paroxetine, escitalopram, and sertraline [11–14].

According to the second perspective, the most used compounds are opioid antagonists, as in the treatment of alcoholism or other forms of addiction. In particular, controlled studies have been conducted for naltrexone and nalmefene on larger samples and with the best results [15, 16].

In the third approach, therapy is based on mood stabilizers such as lithium and atypical antipsychotics, as in the treatment of resistant depression and bipolar disorder [17–19].

### 3.1. Antidepressants

A variety of antidepressant drugs have been studied and tested for the treatment of GD. Controlled clinical trials have shown so far conflicting results.

Hollander et al. (2000) conducted a study on fifteen subjects (ten of them completed the study) with a mean dose of fluvoxamine of 195 mg/day versus placebo. The treatment was administered for 8 weeks for fluvoxamine and for the same length of time for placebo and suggested that fluvoxamine may be effective in the treatment of GD in short-time setting, while the early placebo efficacy appeared to diminish over time [11]. However, Blanco et al. (2002) in a study on thirty-two patients, treated for 6 months in a double-blind trial with placebo and fluvoxamine 200 mg/day, emphasize that the effectiveness of fluvoxamine was not significantly superior to placebo [20]. Another study evaluated topiramate versus fluvoxamine on thirty-one male GD patients, randomized to receive either topiramate or fluvoxamine for 12 weeks. It demonstrated that both topiramate and fluvoxamine monotherapy may be effective in the treatment of GD [21]. Finally, a recent case report showed a positive response to treatment with fluvoxamine in a pathological gambler, observed not only through subjective self-report, but also with fMRI results showing improvements before and after medication in several brain regions [22].

There are currently two conflicting studies about the efficacy of paroxetine in GD. The first one was conducted for 8 weeks on forty-five patients: twenty-three of them were treated with paroxetine (20–60 mg/day) and twenty-two with placebo; paroxetine showed better results than placebo and may therefore be effective in the treatment of gambling disorder [12]. In a second double-blind and placebo-controlled trial, performed on seventy-six patients for 16 weeks, paroxetine did not evidence a statistically significant difference over placebo [23].

Saiz-Ruiz et al. (2005) treated sixty patients with GD diagnosis in a double-blind, placebo-controlled study with flexible doses of sertraline (50 to 150 mg/day) for 6 months. The results demonstrated that the efficacy of sertraline was not significantly superior to placebo in the overall sample [14].

Two studies have tested the efficacy of escitalopram in the treatment of GD. A trial on thirteen gamblers with comorbid anxiety, treated with escitalopram (mean dose 25 mg/day) for 12 weeks in open-label trial and for 8 further weeks in double-blind, placebo-controlled trial, demonstrated significant superiority of escitalopram compared with placebo [13]. The second study was carried out on nineteen subjects treated with flexible doses of escitalopram; both efficacy and a good tolerability of escitalopram were observed [24].

An open-label study on fifteen gamblers treated with citalopram has been published in 2002. The drug appeared to determine a statistically significant improvement on gambling behaviours, depressive symptoms, and quality of life of subjects [25].

A study on thirty-nine patients tested the efficacy of bupropion (mean dose 325 mg/day) versus placebo in the treatment of GD; results indicated that bupropion does not have an efficacy significantly superior to placebo [26]. In contrast, Dannon et al. conducted a randomized trial comparing a group receiving sustained-release bupropion (*N* = 17) with naltrexone-treated group (*N* = 19) for 12 weeks, showing that sustained-release bupropion may be as effective as naltrexone in the treatment of GD [27] (Table 1).

### 3.2. Opioid Antagonists

A growing interest has been addressed to the opioid system in the treatment of GD. Several studies have been conducted to test the efficacy of opioid antagonists in the treatment of the disorder, showing a reduction of urges to engage in the addictive behaviour and longer periods of abstinence [15, 16, 28]. A genetic predisposition has been hypothesized to regulate response to opioid antagonists across diagnostic groups and a family history of alcoholism was associated with positive treatment response to these drugs [24]_._

In a 12-week double-blind, placebo-controlled study on forty-five GD subjects, naltrexone (*μ* opioid receptor antagonist), commonly used in the treatment of alcohol dependence, showed efficacy for a mean dose of 188 mg/day; effectiveness was higher in subjects with more pronounced impulsivity at baseline [15]. In a subsequent 18-week, double-blind, placebo-controlled trial, Grant et al. evaluated the safety and efficacy of 3 different doses of oral naltrexone (50 mg/day, 100 mg/day, or 150 mg/day) in the treatment of GD. Results showed a significantly greater improvement on all variables for the subjects assigned to naltrexone groups, without significant differences between the various doses [29]. The efficacy of naltrexone as a treatment for gambling disorder was also evaluated by a randomized, double-blind, placebo-controlled trial on fifty-two subjects that received an 11-week treatment with a mean dose of 59 mg/day; in this study, no significant differences between the naltrexone and placebo groups were found [30]. Furthermore, Rosenberg et al. recently treated seventy-eight patients with four different psychotropic drugs (naltrexone, topiramate, bupropion, and escitalopram) for two years, with an additional two-year follow-up with no medications; results showed a significant improvement in all groups with a predominant effectiveness for patients treated with naltrexone[31]. Finally, Porchet et al. randomized sixty-two patients to receive oral doses of naltrexone (50 mg/day), haloperidol (2 mg/day), or placebo, demonstrating that naltrexone is functionally more active on the modulation of gambling distortions compared to both haloperidol and placebo [32].

Nalmefene, another opioid antagonist, has shown promising results in the treatment of GD as well. A 16-week, randomized, dose-ranging, double-blind, placebo-controlled trial was conducted randomly assigning two hundred and seven patients to a nalmefene (25 mg/day, 50 mg/day, or 100 mg/day) or placebo treatment group. Subjects who received nalmefene had a statistically significant reduction in the severity of GD [16]. In another randomized study lasting 15 weeks on two hundred and thirty-three patients treated with nalmefene (20 or 40 mg/day) or placebo, nalmefene failed to show statistically significant differences on primary and secondary outcomes. Post hoc analyses of participants who received a full titration of the medication for at least 1 week demonstrated instead that nalmefene 40 mg/day had a significantly greater reduction on the primary outcome measure. These findings suggest that medication dosing may be an important issue to consider in order to achieve symptoms control [33].

Finally, a double-blind and placebo-controlled trial by Grant et al. was conducted on two hundred and eighty-four subjects, treated either for 16 weeks with nalmefene (50–100 mg/day) or for 18 weeks with naltrexone (100–150 mg/day); results showed that a family history of alcoholism appeared to predict response to opiate antagonists in GD [28] (Table 2).

### 3.3. Mood Stabilizers

Mood stabilizers showed anti-impulsive properties as well as efficacy in reducing craving and preventing relapse in different substance-related disorders. Several studies have been conducted to evaluate their usefulness in the treatment of GD, also in bipolar spectrum [34].

Forty pathological gamblers with comorbid bipolar spectrum disorders were evaluated for 10 weeks in a randomized, double-blind, placebo-controlled trial with sustained-release lithium carbonate (mean dose 1170 mg/day), showing that lithium may be an effective treatment in reducing both gambling behaviour and affective instability [17]. Another study on nonbipolar pathological gamblers has highlighted the efficacy of both lithium and valproate in the treatment of PG on forty-two subjects treated in single-blind trial with either lithium (*N* = 23) or valproate (*N* = 19) [35]. A study in 2008 observed by means of baseline PET scans twenty- one GD patients with a comorbid bipolar spectrum diagnosis lifetime. Sixteen of them entered a randomized double-blind placebo-controlled parallel group design trial with lithium and received follow-up PET scans for 10 weeks. The results indicated that cortical areas (orbitofrontal cortex and medial frontal cortex) implicated in impulse control disorders showed an increase in relative glucose metabolic rates (rGMR) in pathological gamblers at baseline; lithium treatment, while alleviating the symptoms, further increased rGMR in these areas [36].

A prospective work published in 2008 has tested efficacy and tolerability of extended-release carbamazepine in eight GD subjects treated for 10 weeks; results suggested that extended-release carbamazepine might be effective in treatment of gambling disorder [37].

Finally, in a recent study by Berlin et al. GD patients were randomized to assume either topiramate 25–300 mg/day (*N* = 20) or placebo (*N* = 22) in a 14-week, double-blind, placebo-controlled, parallel-group trial. The results failed to show any significant effect for topiramate on the primary or secondary outcome measures [38].

### 3.4. Atypical Antipsychotics

Several studies have analysed efficacy of olanzapine in the treatment of GD. In a study by McElroy et al. on forty-two patients, in a double-blind, placebo-controlled trial (*N* = 21) with flexible doses of olanzapine 2.5–15 mg/day (*N* = 21), the latter was not superior to placebo in the short-term treatment of GD symptoms [19]. Another double-blind, placebo-controlled trial lasting 7 weeks on twenty-one pathological gamblers demonstrated that a treatment with olanzapine (mean dose 2.5–10 mg/day) was not associated with better results than placebo [18] (Table 3).

### 3.5. Other Pharmacological Agents

Other pharmacological agents have been tested for the treatment of GD.

Experimental evidence indicated common neurochemical substrates for GD and psychostimulants addiction. Therefore, drugs acting on psychostimulants addiction and impulsive symptoms may also be effective in impulsive GD patients [39]. The atypical stimulant modafinil reduces cocaine abuse and impulsivity in patients with ADHD [40, 41]; the study of Zack and Poulos, a placebo-controlled double-blind trial, tried to determine if modafinil (mean dose 200 mg/day) reduces the reinforcing effects of slot machine gambling in GD subjects, and if this effect is stronger in high versus low impulsivity subjects (*N* = 20). The results showed that modafinil had bidirectional effects in the two groups [42]. The same sample of patients was reevaluated in a prospective study, with clinical results highlighting that modafinil may discourage pathological gamblers from chasing losses but also encourage them to continue betting, rather than quitting while being ahead [43].

A previous research of 2004 suggested that gambling induces effects that closely resemble psychostimulant drug effects; the study, a placebo-controlled trial with D-amphetamine (AMPH) 30 mg/day in gamblers (*N* = 10), comorbid gambler-drinkers (*N* = 6), drinkers (*N* = 8), and healthy controls (*N* = 12), showed that psychostimulants are an important component of gambling addiction [44].

Manipulation of glutamatergic neurotransmission is a relatively young but promising avenue for the development of improved therapeutic agents for the treatment of addiction disorders. Growing evidence has accumulated indicating that ligands acting on glutamatergic transmission are also of potential utility in the treatment of drug addiction, as well as GD [45, 46].

N-acetyl cysteine (NAC), an amino acid that seems to restore glutamate concentration in the nucleus accumbens, proved effective in reducing gambling urges and behaviour in a double-blind, controlled placebo study on twenty-seven GD subjects, highlighting an effectiveness in 59.3% of subjects [47].

In another open-label study on twenty-nine subjects, memantine (D-aspartate N-methyl receptor antagonist which seems to reduce the excitability of glutamate and improve impulsivity) was used, resulting in a reduction of gambling behaviours and an improved cognitive flexibility [48].

Given its dopaminergic and glutamatergic properties, amantadine has been studied in the treatment of GD. A double-blind study on 17 GD patients with Parkinson's disease, randomly assigned to a therapy with amantadine 200 mg/day or placebo, demonstrated that the drug might be useful for the treatment of GD [49]. In addition, a recent case report studied amantadine benefits (doses of 50–150 mg/day) on GD, with a reduction of 43–64% on gambling severity [50].

Acamprosate is derived from homotaurine, a nonspecific GABA agonist, and appears to work by promoting a balance between the excitatory and inhibitory neurotransmitters. Contrasting results have been reported on its use in GD treatment [51, 52]. In an 8-week, open-label trial, twenty-six patients received acamprosate at a dose of 1,998 mg/day. It significantly improved PG-YBOCS, G-SAS scores, and number of gambling episodes [49].

Instead, in a double-blind study of seventeen patients evaluated in order to test baclofen (mean dose of 30 mg/day) and acamprosate (mean dose of 666 mg/day) for GD treatment, results did not show any change in the occurrence of gambling behaviours for both medications [50].

## 4. Conclusions

Currently available data suggest the efficacy of different therapeutic strategies in the treatment of GD, showing that pharmacological research on this disorder may be promising. Although studies indicate some effectiveness of the three main classes of pharmacological interventions (antidepressants, opiate antagonists, and mood stabilizers), future investigations should be addressed to detect differences in outcome among specific subgroups of GD patients. While empirically validated treatments for GD have varying degrees of support, little is known about their mechanisms of action or how specific therapies might work better for specific individuals. In clinical practice, clinicians are accustomed to using combinations of different drugs, in particular to address the comorbid conditions, such as major depression, bipolar disorder, and substance-related disorders [53].

Combination strategies need to be studied, with the goal of providing validated therapeutic algorithms and more effective treatment strategies. In addition, results of published studies refer to a peculiar population of GD patients that requested help and treatment. Moreover, the data on long-term relapse prevention are scarce, compared to that on short-term treatment. Sample size should be expanded and the duration of the studies extended, in order to transfer the data on therapeutic efficacy to a wider population of gamblers and to evaluate the benefits in a longer-term follow-up. Therefore, future studies taking account of these shortcomings will reveal more insight in the underlying mechanisms of GD. Further studies are therefore needed to better understand the mechanisms of action of the different categories of drugs on gambling domains, the appropriate doses for the effective treatment, and the optimal pharmacological approach for GD.

## Figures and Tables

**Table 1:** Antidepressants in gambling disorder.

Study	Drug tested and mean-range dosage	Study design	Study group and duration	Findings
Antidepressants in GD				
Hollander et al. 2000 [11]	Fluvoxamine (SSRI) mean dose 195 mg/day	Double-blind cross-over placebo-controlled	15 patients for 16 weeks	Fluvoxamine is superior to placebo
Blanco et al. 2002 [20]	Fluvoxamine (SSRI) mean dose 200 mg/day	Double-blind placebo-controlled	32 patients for 6 months	Fluvoxamine is not significantly superior to placebo
Kim et al. 2002 [12]	Paroxetine (SSRI) 20–60 mg/day	Double-blind placebo-controlled	45 patients for 8 weeks	Paroxetine is superior to placebo
Grant and Potenza 2003 [23]	Paroxetine (SSRI) 10–60 mg/day	Double-blind placebo-controlled	76 patients for 16 weeks	Paroxetine is not significantly superior to placebo
Saiz-Ruiz et al. 2005 [14]	Sertraline (SSRI) 50–150 mg/day	Double-blind placebo-controlled	60 patients for 6 months	Sertraline is not significantly superior to placebo
Grant et al. 2006 [13]	Escitalopram (SSRI) mean dose 25 mg/day	Open-label *for 12 weeks* Double-blind placebo-controlled *for 8 weeks *	13 patients *with comorbid anxiety* for 20 weeks	Escitalopram is superior to placebo
Black et al. 2007 [26]	Bupropion (NDRI) mean dose 325 mg/day	Double-blind placebo-controlled	39 patients for 12 weeks	Bupropion is not significantly superior to placebo

**Table 2:** Opioid antagonists in gambling disorder.

Study	Drug tested and mean-range dosage	Study design	Study group and duration	Findings
Opioid antagonists in GD				
Kim et al. 2001 [15]	Naltrexone mean dose 188 mg/day	Double-blind placebo-controlled	89 patients for 12 weeks	Naltrexone is significantly superior to placebo
Grant et al. 2008 [29]	Naltrexone 50–150 mg/day	Double-blind placebo-controlled	77 patients for 18 weeks	Naltrexone is significantly superior to placebo
Toneatto et al. 2009 [30]	Naltrexone mean dose 59 mg/day	Double-blind placebo-controlled	52 patients for 11 weeks	Naltrexone is not significantly superior to placebo
Grant et al. 2006 [16]	Nalmefene 25–100 mg/day	Double-blind placebo-controlled	207 patients for 16 weeks	Nalmefene is significantly superior to placebo
Grant et al. 2010 [33]	Nalmefene 20–40 mg/day	Single-blind *for 1 week with placebo* Double-blind placebo-controlled *for 15 weeks *	233 patients for 16 weeks	Nalmefene 40 mg/day is significantly superior to placebo

**Table 3:** Mood stabilizers and atypical antipsychotics in gambling disorder.

Study	Drug tested and mean-range dosage	Study design	Study group and duration	Findings
Mood stabilizers and atypical antipsychotics in GD				
Hollander et al. 2005 [17]	Lithium carbonate mean dose 1170 mg/day	Double-blind placebo-controlled	40 patients *with bipolar comorbid* for 10 weeks	Lithium is significantly superior to placebo
Berlin et al. 2013 [38]	Topiramate 25–300 mg/day	Double-blind placebo-controlled	42 patients for 14 weeks	Topiramate is not significantly superior to placebo
McElroy et al. 2008 [19]	Olanzapine 2.5–15 mg/day	Double-blind placebo-controlled	42 patients for 12 weeks	Olanzapine is not significantly superior to placebo
Fong et al. 2008 [18]	Olanzapine 2.5–10 mg/day	Double-blind placebo-controlled	23 patients for 7 weeks	Olanzapine is not significantly superior to placebo
